# The expanding spectrum of rare monogenic autoinflammatory diseases

**DOI:** 10.1186/1750-1172-8-162

**Published:** 2013-10-16

**Authors:** Isabelle Touitou, Caroline Galeotti, Linda Rossi-Semerano, Véronique Hentgen, Maryam Piram, Isabelle Koné-Paut

**Affiliations:** 1 Laboratoire de génétique des maladies rares et auto-inflammatoires, CHRU de Montpellier, Montpellier, France; 2 INSERM U844, Montpellier, France; 3 Université Montpellier 1, UM1, Montpellier, France; 4 Service de pédiatrie générale et rhumatologie pédiatrique, APHP, CHU de Bicêtre, Université de Paris Sud, Le Kremlin-Bicêtre, France; 5 Service de pédiatre, CH de Versailles, Versailles, France

**Keywords:** Autoinflammatory diseases, Histiocytosis and lymphadenopathy syndrome (H Syndrome), NLRP12 Associated Periodic Syndrome (NAPS12), Deficiency of Interleukin 1 Receptor Antagonist (DIRA), Deficiency of interleukin 36 receptor antagonist (DITRA), Pityriasis rubra pilaris (PRP), Disseminated Superficial Actinic Porokeratosis (DSAP), Autoinflammation LipoDystrophy and Dermatosis syndrome (ALDD)

## Abstract

Monogenic autoinflammatory diseases are a group of hereditary disorders characterized by a clinical and biological inflammatory syndrome in which there is little or no evidence of autoimmunity. The discovery of the first causative gene in 1997 was rapidly followed by the identification of many others from the same group. The mutated proteins can be directly or indirectly involved in the regulation of inflammation. The available literature includes numerous reviews, which address the principle diseases, but we wanted to focus on the most recent rare syndromes. A comprehensive review is thus provided, including taxonomic, genetic, and epidemiological data, along with characteristics defining positive and differential diagnoses and treatment. We believe that this update will assist physicians in correctly naming their patient’s illness. This is an essential step for the effective and targeted management of an orphan disease.

## Abstract (fr)

**Abstract in french:**

Les maladies autoinflammatoires monogéniques sont un groupe de pathologies héréditaires caractérisées par un syndrome inflammatoire clinique et biologique, dans lesquelles on ne retrouve pas ou peu de signe d'autoimmunité. La découverte du premier gène en 1997 a été rapidement suivie par l’identification de nombreux autres appartenant au même groupe. Les protéines mutées participent directement ou indirectement à la régulation de l’inflammation. De nombreuses revues balayant les principales entités sont disponibles dans la littérature, mais nous avons voulu mettre la lumière sur les formes rares les plus récentes. Nous proposons une revue exhaustive incluant des données taxonomiques, génétiques, épidémiologiques, ainsi que des éléments cliniques de certitude et différentiels, et de traitement. Nous espérons que cette mise à jour aidera le médecin à mettre un nom sur la maladie dont souffrent leurs patients atteints de maladie autoinflammatoire, une étape indispensable pour une prise en charge efficace et ciblée.

## Introduction

Autoinflammatory diseases arise from disorders of the innate immune system [1]. The prototype disease is familial Mediterranean fever (FMF), which belongs to the group of hereditary recurrent fevers. Periods of inflammation are predominantly triggered following unnoticed proinflammatory signals. The levels of serum markers of inflammation such as C-reactive protein are always increased during attacks, however autoantibodies remain mostly undetectable. Since the discovery in 1997 of the gene responsible for FMF [2,3], dramatic insights have been gained through establishing the central role of the inflammasomes by unraveling their components and interacting cellular partners [4-6]. Subsequent to the identification of causal mutations in monogenic autoinflammatory diseases, several sequence variants in the causative genes were found to act as susceptibility factors in the more common multifactorial diseases such as rheumatoid arthritis or multiple sclerosis [7]. More recently, monogenic autoinflammatory disorders expanded to include the so far-called inflammatory skin diseases without systemic involvement. Enhanced knowledge relating to the critical physiological mechanisms altered in these diseases fuelled the development of novel, dramatically effective targeted biological drugs such as Interleukin-1 inhibitors [8]. As autoinflammatory diseases are rare, a national referral center nominated by the French ministry of health was set-up (CeRéMAI; [http://asso.orpha.net/CEREMAI/EN/index.html]) in order to improve care for patients. This referral center created Infevers [9] [fmf.igh.cnrs.fr/infevers/], a dedicated online database for autoinflammatory mutations [9].

This review focuses on monogenic autoinflammatory diseases identified within the last five years, including epidemiological and genetic data (Table 1), an exhaustive description of the corresponding disease name and synonyms where available, OMIM and Orphacode numbers, main clinical signs and therapeutic approaches (Table 2).

**Table 1:** Epidemiology and genetics of rare recently recognized monogenic autoinflammatory diseases

**Year of gene discovery**	Disease*		N patients**	**M/F ratio**	Ethnicity***	**Ref**	**Mode of inheritance**	Gene****		**Chromosome location**	**Most frequent mutation**
2008	H Syndrome	Histiocytosis lymphadenopathy plus syndrome	4	2/2	1 Indian case	12	Recessive	*SLC29A3*	Solute Carrier Family	10q22.1	ND
					1 Pakistani case	13					
					1 Morocan case	10			29 (Nucleoside Transporter), Member 3		
					1 Tunisian case	11					
2008	NAPS12	NLRP12 Associated Periodic Syndrome	19	13/6	2 Guadeloupean families	18	Dominant	*NLRP12*	NLR pyrin domain containing protein 12	19q43.42	c.1054C>T, p.Arg352Cys
						17					
					1 Italian family	58					
					6 Eastern-European cases	16					
					1 Armenian case	16					
					1 Italian case	9					
					1 Guadeloupean case	9					
					1 Irish case						
2009	DIRA	Deficiency of Interleukin 1 Receptor Antagonist	17	9/8	1 Turkish family	23	Recessive	*IL1RN*	Interleukin 1	2q13	c.-64_1696del, p.IL1F9_IL1RNdel
					1 Lebanese	22			receptor antagonist		
					family	22					
					2 Dutch families	22					c.213_227del, p.Asp72_Ile76del
					1 Dutch case	22					
					1 Canadian case	22,27					
					2 Puerto-Rican cases	25					
					2 Brazilian cases	24,26					
					2 Unknown						
					3 Dutch,						
					1 Lebanese,						
					1 Turkish,						
					1 from unknown ancestry						
2011	DITRA	Deficiency of Interleukin	66	26/37	3 Tunisian families	30	Recessive	*IL36RN*	Interleukin 36 receptor antagonist	2q13	c.80C>T, p.Leu27Pro
					1 Algerian family	9					
		36 Receptor Antagonist			19 European cases	32,35,37					c.338C>T, p.Ser113Leu
					10 Asian cases	35					c.28C>T, p.Arg10*
					6 Tunisian cases	30					
					6 German cases	36					
					3 Japanese cases	33,34					
					1 Spanish case	9					
					1 Turkish case	36					
					1 Iraqi case	36					
					1 Russian case	37					
2012	PSORS2	Psoriasis susceptibility 2	45	24/21	1 European family	42	Dominant	*CARD14*	Caspase recruitment domain-containing protein 14	17q25.3	c.349G>A, p.Gly117Ser
					1 Taiwanese family	42					
					1 Tunisian family	43					
					3 German cases	36					
	PRP	Pityriasis Rubra Pilaris	17	9/8	4 Israeli families	41					
2012	DSAP	Disseminated Superficial Actinic Porokeratosis	>45	21/16/ND	20 Chinese families,	48.49	Dominant	*MVK*	Mevalonate Kinase	12q24	C.604G>A
											p.Gly202Arg
					6 Chinese cases						
2010	ALDD (JMP, NNS, CANDLE)	Autoinflammation, lipodystrophy, and dermatosis syndrome	40	26/14	22 Japanese families	53,57	Recessive	*PSMB8*	Proteasome Subunit,	6p21.32	c.224C>T, p.Thr75Met
					1 Portuguese family	56			Beta-Type, 8		c.602G>T,
					1 Mexican family	56					p.Gly201Val
					3 Hispanic cases	54					
					3 Spanish cases	54					
					2 American cases	54					
					1 Japanese case	53					
					1 Israeli case	54					
					1 Italian case	9					
2012	APLAID	Autoinflammation, Antibody deficiency, and Immune Dysregulation syndrome	2	1/1	1 family of unknown origin	14	Dominant	*PLCG2*	Phospholipase C, gamma 2	16q23.3	ND
2012	NA	Autoinflammatory syndrome with pyogenic bacterial infection and amylopectinosis	3	1/2	1 French family	15	Recessive	*RBCK1*	RANBP-Type and C3HC4-Type Zinc Finger-Containing 1	20p13	ND
					1 Italian case						

* Commonly used disease names as defined in Table 2 ** Genetically confirmed. *** “Family” refers to multiplex families; “Case” refers to sporadic patients **** Approved HUGO.

NA: Not assigned yet. ND: Not determined.

**Table 2:** Clinical features of rare recently recognized monogenic autoinflammatory diseases

**OMIM**	**ORPHA-CODE**	**Disease acronyme**	Disease extended name*	**Synonyms**						**Age at onset**	**Key symptoms**	**Differential diagnosis**	**Treatment**
612373	168569 254707 254712 254723	**H Syndrome**	Histiocytosis-lymphadenopathy plus syndrome	PHID	Pigmentary Hypertrichosis and non-autoimmune Insulin-dependent Diabetes mellitus	FHC	Faisalabad Histiocytosis	SHML	Sinus Histiocytosis with Massive Lymphadenopathy	Infancy	Histiocytosis	FCAS2	NSAID (IL-1 and TNF blockades not effective)
												FCAS3	
												ALDD	
												Other causes of insulin-dependent diabetes	
611762	247868	FCAS2	Familial Cold Autoinflammatory Syndrome 2	**NAPS12**	NLRP12 Associated Periodic Syndrome	**NLRP12AD**	NLRP12-associated disorder			Infancy Childhood Adulthood	Urticaria, fever, myalgia, arthralgia	FCAS1 FCAS3	NSAID (IL-1 blockades not effective)
612852	210115	OMPP	Osteomyelitis, sterile Multifocal, with Periostitis and Pustulosis	**DIRA**	Deficiency of Interleukin 1 Receptor Antagonist					Neonatal	Neutrophilic pustular dermatosis, periostitis, aseptic multifocal osteomyelitis	PSORP Other dermatologic and infectious conditions	IL1 blockades (NSAID not effective)
614204	247353	PSORP	Pustular Psoriasis, Generalized	**DITRA**	Deficiency of Interleukin 36 Receptor Antagonist	GPP	Generalized Pustular Psoriasis			Infancy, Childhood, Adulthood	Diffuse erythematous pustular rash, fever, malaise and diffuse pain, systemic inflammation	OMPP Other autoinflammatory diseases	NSAID, Vitamin D3, Acitretin, TNF and IL-1 blockades
602723	NA	PSORS2	**Psoriasis susceptibility 2**							Variable	Round, well circumscribed erythematous plaques covered by a thick silver scale with a predilection for elbows, knees, scalp, lumbosacral and anogenital regions	Other papulosquamous disorders	Corticosteroids, calcineurin inhibitor, calcipotriene, emollients, keratolytic agents, ultraviolet light, retinoids, methotrexate, cyclosporine, anti-TNF agents
173200	2897	**PRP**	Pityriasis Rubra Pilaris							Neonatal Early childhood	Small keratotic follicular papules, disseminated salmon-colored scaly plaques surrounding islands of normal skin, diffuse red-orange palmoplantar keratoderma	Phrynoderma (vitamin A deficiency), psoriasis, erythrokeratodermia, other causes of cornification	Emollients, topical corticosteroids, tazarotene, keratolytic agents, calcineurin inhibitor, systemic retinoids, TNFα blocking agents
175900	79152	POROK3	Porokeratosis 3, Disseminated Superficial Actinic Type	**DSAP**	Disseminated Superficial Actinic Porokeratosis					Adult	UV sensitive, Epidermal cornification, round and brownish lesions	Neoplastic or hyperplastic squamous proliferations	Cryotherapy, topical reagents, electrodessication, laser ablation, photodynamic therapy
256040	2615	ALDD	Autoinflammation, LipoDystrophy, and Dermatosis syndrome	**JMP**	Joint contractures, muscle atrophy, Microcytic anemia, and Panniculitis-induced lipodystrophy syndrome.	**NNS**	Nakajo-Nishimura Syndrome	**CANDLE**	Chronic Atypical Neutrophilic Dermatosis with Lipodystrophy and Elevated temperature syndrome	Neonatal	Fever, skin rash, panniculitis, lipoatrophy	Still’s disease, CINCA, mucopolysaccharidosis, lupus, dermatomyositis, laminopathies, Aicardi Goutieres syndrome	NSAID, Interferon γ, JAK inhibitors?
614878	324530	**APLAID**	Autoinflammation, antibody deficiency, and immune dysregulation syndrome							ND	Neutrophilic skin lesions, IBD, recurrent sino pulmonary infections	Other immunodeficiencies, IBD, PLAID	ND
NA	329173	NA	Autoinflammatory syndrome with pyogenic bacterial infection and amylopectinosis							ND	ND	ND	ND

*Approved OMIM; NA Not assigned yet; ND: too few patients to delineate clear criteria. Commonly used disease names are in bold text.

NSAID: nonsteroidal anti-inflammatory drugs.

CINCA: Chronic, Infantile, Neurologic, Cutaneous and Articular syndrome, IBD: inflammatory bowel disease, PLAID: PLCg2-Associated antibody Deficiency.

### Rare recently recognized monogenic autoinflammatory diseases

The correct diagnosis of monogenic autoinflammatory diseases relies on the physicians’ awareness. Due to the rarity of these disorders, a very low number of cases are reported. For example, less than five patients with clear autoinflammatory features and causative mutations have been published for each of H syndrome [10-13], Autoinflammation and PLCG2-associated antibody deficiency and Immune Dysregulation syndrome (APLAID) [14], and RANBP-Type and C3HC4-Type Zinc Finger-Containing 1 (RBCK1)-related disease [15]. These three conditions are thus cited in Tables 1 and 2 only. Moreover, as mutations in these rare conditions have apparently systemic and pleiotropic effects, the patients’ clinical spectrum is heterogeneous and not yet completely delineated. Molecular analysis of the candidate genes may also fail to detect mutations. Consequently, the frequency of these diseases is unknown and too little feedback and data exist on the clinical significance and penetrance of the variants to ensure adequate genetic counseling for these patients.

### NLRP12 Associated Periodic Syndrome (NAPS12)

This disease is characterized by recurrent periods of fever, combined with various systemic manifestations such as myalgia, arthralgia, headaches and skin urticarial-like rash although some patients may display arthralgia or myalgia only [16-18]. The most severely affected patients suffer from neurosensory signs (headache, deafness) [18]. The inflammatory attacks last 5-10 days and are triggered by physical exertion or cold exposure. These attacks can be very disabling in everyday life and can have a major impact on school attendance or work. The disease appears to be more active in children and teenagers than in adults.

Treatment is not standardized and no long-term medication has proven to be effective. Patients with a mild phenotype may be treated symptomatically with non-steroidal anti-inflammatory drugs. There is currently no therapeutic option available for patients with the most severe phenotype, and long-term treatment with interleukin (IL)-1 inhibitors (Anakinra, Kineret®) failed in the two cases tested [19].

The genetic defect of this disease was identified in 2008 [18] as a mutation in the gene encoding NLR pyrin domain-containing protein 12 (*NLRP12*) belonging to the intracellular Nod-like receptor (NLR) family. This protein is an intracellular sensor of the innate immune system, that regulates inflammatory processes through inhibition of nuclear factor kappa B (NF-κB) [18] and IL1β [16] signaling. *NLRP12* mutations are likely responsible for a loss of protein function resulting in upregulation of this pro-inflammatory pathway. However, the exact role of NLRP12 remains unknown. It may participate in the formation of an NLRP3-independent inflammasome and be crucial for the recognition of intracellular pathogens. NLRP12 could also play an important role in the homeostasis of the gut by controlling the genesis of inflammatory diseases of the gastrointestinal tract and/or colorectal cancer [20,21]. Two unambiguous mutations, c.850C > T, p.Arg284*; c.2072 + 2dupT, p.Val635Thrfs*12 [18] as well as two missense substitutions c.1054C > T, p.Arg352Cys [16] and c.882C > G, p.Asp294Glu [17] were proven pathogenic through functional studies.

### Deficiency of Interleukin 1 Receptor Antagonist (DIRA)

Sterile multifocal osteomyelitis with periostitis and pustulosis (OMPP) is more commonly referred to as DIRA [22]. DIRA is an early-onset auto-inflammatory disease that presents with neutrophilic pustular dermatosis, periostitis, aseptic multifocal osteomyelitis, and high acute-phase reactants. Patients develop pustular skin rashes, gastrointestinal reflux, and multifocal osteomyelitis during the neonatal period. One patient presented with early onset intrauterine disease and intrauterine fetal demise [23]. Massive systemic inflammatory syndrome resulting in patient death has been described [22]. Other manifestations include osteopenia, interstitial pneumonia often causing hypoxemia and dyspnea or localized ground-glass opacities and areas of atelectasis at chest computed tomography, vesicular stomatitis, mouth ulcers, ribs widening, periosteal reaction, joint swelling, cervical vertebra fusion, hepatosplenomegaly, thrombosis and vasculopathy [22,24-26]. Skin biopsies show neutrophilic epidermal and dermal infiltration, concomitant superficial folliculitis, with subcorneal localization of the pustules. Differential diagnosis includes other autoinflammatory conditions especially deficiency of the Interleukin 36 receptor antagonist (DITRA), dermatologic conditions (infantile pustular psoriasis, tinea, bacterial folliculitis, scabies, eosinophilic pustular folliculitis, erythema toxicum neonatorum, transient neonatal pustular melanosis…), and infectious diseases (arthritis, osteomyelitis).

Antibiotics and conventional disease-modifying antirheumatic drugs including steroids are of limited benefit, however blocking IL-1 signaling with anakinra dramatically improved clinical symptoms within days, resolving osteolytic lesions, normalizing acute-phase reactants, and permitting appropriate growth [22,24-29]. Patients with DIRA need to remain on lifelong IL-1 inhibitory therapy. Previous attempts at stopping or weaning anakinra have resulted in disease flares.

The disease is caused by mutations of the gene encoding the protein IL1RA. This protein is a natural antagonist of the potent proinflammatory interleukins-1 cytokines and mutations result in over activity of IL-1α as well as IL-1beta is expected in case of IL-1RA deficiency Two founder deletions were reported, one Puerto Rican: c.-64_1696del, p.IL1F9_IL1RNdel [22,24,27], and one Brazilian: c.213_227del, p.Asp72_Ile76del [25].

### Deficiency of IL-36 receptor antagonist (DITRA)

Interleukin-36-receptor antagonist deficiency is a hereditary auto-inflammatory disease characterized by repeated flares of generalized pustular psoriasis (GPP). The flares include sudden diffuse pustular erythematous rash associated with high fever, general malaise, systemic inflammation and sometimes ^'^geographic tongue’ and nail dystrophy [30]. Organ inflammation such as cholangitis can occur during the disease course. In most patients, symptoms develop during childhood though the age of onset can vary considerably. Neonatal onset has also been reported. Failure to thrive may occur. Elevated C-reactive protein serum levels and marked leukocytosis are constant during disease attacks. Differential diagnosis includes other causes of GPP, mainly DIRA [22], in cases where symptoms appear during the first weeks of life.

There is no established treatment. Topical and oral steroids, vitamin D3, acitretin and anti-TNF-α, are effective. Our group has recently successfully treated an infant with DITRA not responding to topical and oral steroids or to acitretine with anakinra [31].

IL-36 receptor antagonist (IL-36RA) is a natural antagonist of IL-36α, IL-36β and IL-36γ, three cytokines belonging to the IL-1 family. Mutations result in decreased IL-36RA antagonistic effects due to a reduction of both IL-36RA protein stability and affinity for its receptor, the consequence of which is uncontrolled inflammation [30,32-37]. The high resulting expression of IL-36R and of the three agonists in epithelial tissues like skin likely causes GPP, the main clinical feature in all DITRA patients. In a recent study, Setta-Kafetzi et al. identified IL36RN variant alleles in palmarplantar pustulosis and acrodermatitis continua of hallopeau, two forms of GPP genetically distinct from psoriasis vulgaris [35].

Among the most frequent genetic alterations are one Tunisian founder missense mutation: c.80C > T, p.Leu27Pro1; one European recurrent missense mutation: c.338C > T, p.Ser113Leu2; and one Japanese founder nonsense mutation: c.28C > T, p.Arg10*3.

### Psoriasis susceptibility (PSORS)2 and pityriasis rubra pilaris (PRP)

Psoriasis is a common and chronic immune-mediated inflammatory disease of the skin affecting approximately 2% of individuals of European descent with a strong genetic susceptibility [38]. Classic lesions consist of round, well-circumscribed erythematous plaques covered by a thick silver scale with a predilection for elbows, knees, scalp, lumbosacral and anogenital regions. Fingernail plate involvement includes pitting and distal onycholysis. Inverse psoriasis (flexural distribution), guttate psoriasis, pustular or erythrodermic psoriasis are other clinical variants. Pityriasis rubra pilaris (PRP) is a chronic skin disorder of abnormal keratinization classified by Griffiths into five types based on the age of onset and the clinical features [39]. Familial PRP belongs to the type V group (atypical juvenile) and accounts for about 5% of all cases. This chronic skin disorder usually presents at birth or appears during early childhood. It is characterized by small keratotic follicular papules forming disseminated salmon-colored scaly plaques that surround islands of normal skin, and diffuse red-orange palmoplantar keratoderma. Lesions typically progress rostral to caudal. While nails can be dystrophic, pitting of nails is not a feature of this disease. Skin biopsies can be performed if the clinical features or the responses to treatment are unusual. Histopathological analysis shows psoriasiform hyperplasia presenting as acanthosis with elongation of the rete ridges, parakeratosis and mononuclear cell infiltrate. Compared to psoriasis, the epidermal hyperplasia in PRP is more irregular, the granular layer is preserved, follicular plugging is characteristic and neutrophils in the stratum corneum are not essential. Differential diagnosis of psoriasis includes other papulosquamous disorders. PRP shows clinical features similar to phrynoderma (vitamin A deficiency), psoriasis, erythrokeratodermia and other causes of cornification.

Management of psoriasis includes patient education, topical therapy (corticosteroids, calcineurin inhibitor, calcipotriene, emollients, keratolytic agents), ultraviolet light, and systemic therapy (retinoids, methotrexate, cyclosporine and anti-TNF agents). The treatment of PRP is often disappointing. Milder cases of the disease can be treated with emollients, topical corticosteroids, tazarotene, keratolytic agents or calcineurin inhibitor whereas systemic retinoids and/or TNFα blocking agents are required in severe cases.

The prognosis of both disorders is that of most chronic diseases, with spontaneous remissions and exacerbations. Arthritis is present in about one fourth of psoriasis patients, and there is an increased risk of metabolic syndromes and cardiovascular diseases [40].

Both phenotypes have been associated to CAspase Recruitment Domain-containing protein 14 (*CARD14*) mutations [36,41-43]. CARD14 is an epidermal regulator of NF-κB transcription factor. Mutations in CARD14 initiate a process that includes inflammatory cell recruitment by keratinocytes and perpetuate a vicious cycle of epidermal inflammation and regeneration that cause the abnormal keratinization. More specifically, c.349G > A, p.Gly117Ser (the most prevalent mutation), and c.413A > C, p.Glu138Ala (a *de novo* mutation) were shown to lead to enhanced NF-κB activation and upregulation of a subset of psoriasis-associated genes in keratinocytes [43].

### Disseminated Superficial Actinic Porokeratosis (DSAP)

This disease described in 1966 by Chernosky [44] represents the most common form of porokeratosis, a group of diseases characterized by epidermal cornification with epidermal cell hyperproliferation and premature apoptosis of keratinocytes. Triggers include ultraviolet light exposure and immunosuppression. Multiple small (0.5-1 cm) round and brownish lesions surrounded by a hyperkeratotic rim develop on sun-exposed areas of the skin particularly on the lower legs and forearms. Lesions usually appear in summer and improve or disappear during winter. The disease usually starts during the third or fourth decades of life, and rarely affects children. While it is usually benign, squamous cell carcinoma or Bowen’s disease may occasionally develop within the lesions.

Histopathological examination of the skin sampled from the edge of the peripheral rim characteristically shows an angulated cornoid lamella and a parakeratotic column overlying an area of epidermis with an absent or reduced granular layer [45]. Differential diagnosis includes neoplastic or hyperplastic squamous skin lesions.

Cryotherapy, topical reagents (retinoids, imiquimod, 5-fluorouracil, keratolytic), electrodessication, laser ablation or photodynamic therapy are used to destroy the abnormal keratinocyte clones [46].

The mode of inheritance is dominant however many sporadic cases of porokeratosis have been described [47]. Mutations in the MeValonate Kinase (*MVK*) gene were found in DSAP patients [48,49]. The MVK enzyme is involved in the biosynthesis of cholesterol and isoprenoid. It is also thought to play a role in regulating calcium-induced keratinocyte differentiation and could protect keratinocytes from apoptosis induced by type A ultraviolet radiation. All genetically confirmed patients are of Chinese origin. Fourteen different mutations have been found, among which, 10 accounted for one third of the familial cases. Mutations in this gene were initially demonstrated as responsible for recessive complete (mevalonic aciduria [50], OMIM 610377) and incomplete (hyperIgD syndrome [51,52], OMIM 260920) mevalonate kinase deficiency.

### Autoinflammation, LipoDystrophy, and Dermatosis syndrome (ALDD)

ALDD is a systemic inflammatory condition starting within the first months of life, and comprising elevated fever, panniculitis with lipoatrophy, purplish and swollen eyelids, arthralgia, developmental retardation, and increased acute phase reactants [53,54]. Key symptoms include a persistent fever of >38.5°C, steroid-sensitive erythema nodosum-like (edematous and purpuric) plaques, long clubbed fingers, hyperhidrosis, myositis, hepatosplenomegaly, macroglossia, facial and limbs lipoatrophy, and developmental (height, weight and IQ) retardation. Skin biopsies show immature myeloid-lineage cells with mitoses. Some patients may have joint contracture, auricular and nasal chondritis, and calcification of the basal ganglia. Acute cardiovascular event is the leading cause of death in these patients for whom life expectancy is notably reduced. Differential diagnosis includes other autoinflammatory conditions, in particular Sweet’s syndrome, Still’s disease and Chronic, Infantile, Neurological, Cutaneous and Articular (CINCA) syndrome, storage diseases (mucopolysaccharidosis), lupus, dermatomyositis, laminopathies (progeroid syndromes), and other interferonopathies like Aicardi Goutieres syndrome, an interferon type I related disease. Biological findings include microcytic or normocytic anemia, thrombopenia (rare), mild increase in levels of amino transferase enzymes, perturbation of glucose and lipid metabolism (hyperglycemia and glucose intolerance, increased cholesterol and triglycerides), increased erythrocyte sedimentation rate and γ globulins levels, and some markers of autoimmunity (antinuclear and anti-neutrophilic cytoplasmic auto-antibodies, lupus anticoagulant, positive Coombs test).

Management of these patients is by palliative care**.** The need for steroids is very high even in combination with anti IL-1, anti IL-6 or anti-TNF treatments. Drugs targeting interferon γ are warranted. JAK kinase inhibitors are currently under clinical trials at the National Institute of Health, Bethesda, USA [55].

Most (N = 28) of the reported patients carried homozygous mutations in the Proteasome Subunit, Beta-Type, 8 (*PSMB8*) gene [53,54,56,57]. This gene encodes β5i, a catalytic subunit of the immunoproteasome, an intracellular protease complex specialized for degradation of polyubiquitinated proteins. This ubiquitin-proteasome system degrades unnecessary proteins and regulates the cell cycle, gene repair and nuclear factor NF-κB activation [53]. The mutated protein impairs the assembly of this immunoproteasome. One nonsense (c.405C > A, p.Cys135*) [54] and five missense mutations of which two are recurrent (c.224C > T, p.Thr75Met and c.602G > T, p.Gly201Val) have been reported [9]. As the prognosis is so severe, prenatal diagnosis can be discussed.

## Conclusions

Hereditary recurrent fevers, as well as Majeed syndrome, Blau syndrome, and Pyogenic sterile Arthritis, Pyoderma gangrenosum and Acne (PAPA) syndrome, have been extensively reviewed since the concept of autoinflammation was created in 1999 [58]. However, very little information has been assembled regarding the phenotype, genetics and epidemiology of the more recently discovered autoinflammatory disorders. Here in this review, we aimed to offer novel diagnostic options to those patients for whom a genetic link could not be established. More than 25 autoinflammatory genes were listed during the last international conference on autoinflammation, Lausanne, 23-26 May, 2013, including at least five to six new ones in the pipeline. Both phenotype and genotype heterogeneity have been described which further hinders correct diagnosis and highlights the necessity for a gene and disease nomenclature consensus. A systematic in-depth description of the clinical features of these patients has been undertaken thanks to the Eurofever initiative [59,60]. Massively parallel sequencing approaches should help in identifying the causative gene among those already known or novel genes through whole exome or genome sequencing. Development of such innovative genetic diagnostic tools is in progress through an International network (I Touitou, personal communication).

The correct diagnosis of these diseases is essential for the relevant management of patients affected by rare autoinflammatory diseases as remarkably effective therapies have been developed recently which have the potential to avoid the risk of fatal complications such as amyloidosis, or sensorineural impairment. Rarely emphasized yet equally important is being able, even after many years as in some cases, to name the patient’s disease, which can be extremely useful from a psychological point of view.

## Competing interests

The authors declare that they have no competing interests.

## Authors’ contributions

IT and IKP conceived and designed the manuscript. IKP coordinated and assigned the writing of the different disease items: CG: DIRA; LR: DITRA; IKP: ALDD; VH: NALP12; MP: DSAP and PRP. IT collected the data, assembled the first draft, and polished the manuscript. All authors read the final manuscript and gave approval for the version to be published. All authors read and approved the final manuscript.

## Authors’ information

All authors are members of the French reference centre for autoinflammatory diseases (CeRéMAI, [http://asso.orpha.net/CEREMAI/EN/index.html]) coordinated by IKP. IT developed infevers [http://fmf.igh.cnrs.fr/ISSAID/infevers/], an online registry devoted to autoinflammatory disease mutations.

## References

[B1] Kastner DL Aksentijevich I Goldbach-Mansky R Autoinflammatory disease reloaded: a clinical perspective Cell 2010 8 784 790 20303869 10.1016/j.cell.2010.03.002 PMC3541025

[B2] Consortium TFF A candidate gene for familial Mediterranean fever Nat Genet 1997 8 25 31 10.1038/ng0997-25 9288094

[B3] TheInternationalFMFConsortium Ancient missense mutations in a new member of the RoRet gene family are likely to cause familial Mediterranean fever Cell 1997 8 797 807 10.1016/s0092-8674(00)80539-5 9288758

[B4] Conforti-Andreoni C Ricciardi-Castagnoli P Mortellaro A The inflammasomes in health and disease: from genetics to molecular mechanisms of autoinflammation and beyond Cell Mol Immunol 2011 8 135 145 21258359 10.1038/cmi.2010.81 PMC4003142

[B5] Schroder K Tschopp J The inflammasomes Cell 2010 8 821 832 20303873 10.1016/j.cell.2010.01.040

[B6] Jeru I Amselem S Inflammasome and interleukin 1 Rev Med Interne 2011 8 4 218 224 doi:10.1016/j.revmed.2010.02.013. Epub 2010 Jun 11. French 20541850 10.1016/j.revmed.2010.02.013

[B7] Touitou I Inheritance of autoinflammatory diseases: shifting paradigms and nomenclature J Med Genet 2013 8 349 359 23536687 10.1136/jmedgenet-2013-101577

[B8] Ter Haar N Lachmann H Ozen S Woo P Uziel Y Modesto C Kone-Paut I Cantarini L Insalaco A Neven B Treatment of autoinflammatory diseases: results from the Eurofever Registry and a literature review Ann Rheum Dis 2013 8 5 678 685 doi:10.1136/annrheumdis-2011-201268. Epub 2012 Jun 29 22753383 10.1136/annrheumdis-2011-201268

[B9] Milhavet F Cuisset L Hoffman HM Slim R El-Shanti H Aksentijevich I Lesage S Waterham H Wise C Sarrauste Dementhiere C The infevers autoinflammatory mutation online registry: update with new genes and functions Hum Mutat 2008 8 803 808 18409191 10.1002/humu.20720

[B10] De Jesus J Imane Z Senee V Romero S Guillausseau PJ Balafrej A Julier C SLC29A3 mutation in a patient with syndromic diabetes with features of pigmented hypertrichotic dermatosis with insulin-dependent diabetes, H syndrome and Faisalabad histiocytosis Diabetes Metab 2013 8 281 285 23623699 10.1016/j.diabet.2013.03.007

[B11] Melki I Lambot K Jonard L Couloigner V Quartier P Neven B Bader-Meunier B Mutation in the SLC29A3 gene: a new cause of a monogenic, autoinflammatory condition Pediatrics 2013 8 e1308 e1313 23530176 10.1542/peds.2012-2255

[B12] Mohanan S Chandrashekar L Semple RK Thappa DM Rajesh NG Negi VS Gulati R H syndrome with a novel homozygous R134C mutation in SLC29A3 gene Int J Dermatol 2013 8 820 823 23789599 10.1111/j.1365-4632.2012.05838.x

[B13] Senniappan S Hughes M Shah P Shah V Kaski JP Brogan P Hussain K Pigmentary hypertrichosis and non-autoimmune insulin-dependent diabetes mellitus (PHID) syndrome is associated with severe chronic inflammation and cardiomyopathy, and represents a new monogenic autoinflammatory syndrome J Pediatr Endocrinol Metab 2013 8 6 10.1515/jpem-2013-0062 23729543

[B14] Zhou Q Lee GS Brady J Datta S Katan M Sheikh A Martins MS Bunney TD Santich BH Moir S A Hypermorphic Missense Mutation in PLCG2, Encoding Phospholipase Cgamma2, Causes a Dominantly Inherited Autoinflammatory Disease with Immunodeficiency Am J Hum Genet 2012 8 713 720 23000145 10.1016/j.ajhg.2012.08.006 PMC3484656

[B15] Boisson B Laplantine E Prando C Giliani S Israelsson E Xu Z Abhyankar A Israel L Trevejo-Nunez G Bogunovic D Immunodeficiency, autoinflammation and amylopectinosis in humans with inherited HOIL-1 and LUBAC deficiency Nat Immunol 2012 8 1178 1186 23104095 10.1038/ni.2457 PMC3514453

[B16] Jeru I Le Borgne G Cochet E Hayrapetyan H Duquesnoy P Grateau G Morali A Sarkisian T Amselem S Identification and functional consequences of a recurrent NLRP12 missense mutation in periodic fever syndromes Arthritis Rheum 2012 8 1459 1464 10.1002/art.30241 21538323

[B17] Borghini S Tassi S Chiesa S Caroli F Carta S Caorsi R Fiore M Delfino L Lasiglie D Ferraris C Clinical presentation and pathogenesis of cold-induced autoinflammatory disease in a family with recurrence of an NLRP12 mutation Arthritis Rheum 2011 8 830 839 21360512 10.1002/art.30170 PMC3112487

[B18] Jeru I Duquesnoy P Fernandes-Alnemri T Cochet E Yu JW Lackmy-Port-Lis M Grimprel E Landman-Parker J Hentgen V Marlin S Mutations in NALP12 cause hereditary periodic fever syndromes Proc Natl Acad Sci U S A 2008 8 1614 1619 18230725 10.1073/pnas.0708616105 PMC2234193

[B19] Jeru I Hentgen V Normand S Duquesnoy P Cochet E Delwail A Grateau G Marlin S Amselem S Lecron JC Role of interleukin-1beta in NLRP12-associated autoinflammatory disorders and resistance to anti-interleukin-1 therapy Arthritis Rheum 2011 8 2142 2148 21480187 10.1002/art.30378

[B20] Allen IC Wilson JE Schneider M Lich JD Roberts RA Arthur JC Woodford RM Davis BK Uronis JM Herfarth HH NLRP12 suppresses colon inflammation and tumorigenesis through the negative regulation of noncanonical NF-kappaB signaling Immunity 2012 8 742 754 22503542 10.1016/j.immuni.2012.03.012 PMC3658309

[B21] Zaki MH Vogel P Malireddi RK Body-Malapel M Anand PK Bertin J Green DR Lamkanfi M Kanneganti TD The NOD-like receptor NLRP12 attenuates colon inflammation and tumorigenesis Cancer Cell 2011 8 649 660 22094258 10.1016/j.ccr.2011.10.022 PMC3761879

[B22] Aksentijevich I Masters SL Ferguson PJ Dancey P Frenkel J Van Royen-Kerkhoff A Laxer R Tedgard U Cowen EW Pham TH An autoinflammatory disease with deficiency of the interleukin-1-receptor antagonist N Engl J Med 2009 8 2426 2437 19494218 10.1056/NEJMoa0807865 PMC2876877

[B23] Altiok E Aksoy F Perk Y Taylan F Kim PW Ilikkan B Asal GT Goldbach-Mansky R Sanal O A novel mutation in the interleukin-1 receptor antagonist associated with intrauterine disease onset Clin Immunol 2012 8 77 81 22940634 10.1016/j.clim.2012.08.003 PMC3463868

[B24] Reddy S Jia S Geoffrey R Lorier R Suchi M Broeckel U Hessner MJ Verbsky J An autoinflammatory disease due to homozygous deletion of the IL1RN locus N Engl J Med 2009 8 2438 2444 19494219 10.1056/NEJMoa0809568 PMC2803085

[B25] Jesus AA Osman M Silva CA Kim PW Pham TH Gadina M Yang B Bertola DR Carneiro-Sampaio M Ferguson PJ A novel mutation of IL1RN in the deficiency of interleukin-1 receptor antagonist syndrome: description of two unrelated cases from Brazil Arthritis Rheum 2011 8 4007 4017 22127713 10.1002/art.30588 PMC3463867

[B26] Stenerson M Dufendach K Aksentijevich I Brady J Austin J Reed AM The first reported case of compound heterozygous IL1RN mutations causing deficiency of the interleukin-1 receptor antagonist Arthritis Rheum 2011 8 4018 4022 21792839 10.1002/art.30565

[B27] Minkis K Aksentijevich I Goldbach-Mansky R Magro C Scott R Davis JG Sardana N Herzog R Interleukin 1 receptor antagonist deficiency presenting as infantile pustulosis mimicking infantile pustular psoriasis Arch Dermatol 2012 8 747 752 22431714 10.1001/archdermatol.2011.3208 PMC3474848

[B28] Schnellbacher C Ciocca G Menendez R Aksentijevich I Goldbach-Mansky R Duarte AM Rivas-Chacon R Deficiency of Interleukin-1 Receptor Antagonist Responsive to Anakinra Pediatr Dermatol 2012 doi:10.1111/j.1525-1470.2012.01725.x. [Epub ahead of print] 10.1111/j.1525-1470.2012.01725.x PMC3548019 22471702

[B29] Brau-Javier CN Gonzales-Chavez J Toro JR Chronic cutaneous pustulosis due to a 175-kb deletion on chromosome 2q13: excellent response to anakinra Arch Dermatol 2012 8 301 304 22431772 10.1001/archdermatol.2011.2857 PMC3313085

[B30] Marrakchi S Guigue P Renshaw BR Puel A Pei XY Fraitag S Zribi J Bal E Cluzeau C Chrabieh M Interleukin-36-receptor antagonist deficiency and generalized pustular psoriasis N Engl J Med 2011 8 620 628 21848462 10.1056/NEJMoa1013068

[B31] Rossi-Semerano L Piram M Chiaverini C De Ricaud D Smahi A Kone-Paut I First Clinical Description of an Infant With Interleukin-36-Receptor Antagonist Deficiency Successfully Treated With Anakinra Pediatrics 2013 8 4 e1043 e1047 Epub 2013 Sep 9. 24019411 10.1542/peds.2012-3935

[B32] Onoufriadis A Simpson MA Pink AE Di Meglio P Smith CH Pullabhatla V Knight J Spain SL Nestle FO Burden AD Mutations in IL36RN/IL1F5 are associated with the severe episodic inflammatory skin disease known as generalized pustular psoriasis Am J Hum Genet 2011 8 432 437 21839423 10.1016/j.ajhg.2011.07.022 PMC3169817

[B33] Sugiura K Takeichi T Kono M Ogawa Y Shimoyama Y Muro Y Akiyama M A novel IL36RN/IL1F5 homozygous nonsense mutation, p.Arg10X, in a Japanese patient with adult-onset generalized pustular psoriasis Br J Dermatol 2012 8 699 701 22428995 10.1111/j.1365-2133.2012.10953.x

[B34] Farooq M Nakai H Fujimoto A Fujikawa H Matsuyama A Kariya N Aizawa A Fujiwara H Ito M Shimomura Y Mutation Analysis of the IL36RN Gene in 14 Japanese Patients with Generalized Pustular Psoriasis Hum Mutat 2013 8 176 183 22903787 10.1002/humu.22203

[B35] Setta-Kaffetzi N Navarini AA Patel VM Pullabhatla V Pink AE Choon SE Allen MA Burden AD Griffiths CE Seyger MM Rare Pathogenic Variants in IL36RN Underlie a Spectrum of Psoriasis-Associated Pustular Phenotypes J Invest Dermatol 2013 8 5 1366 1369 doi:10.1038/jid.2012.490. Epub 2013 Jan 10 23303454 10.1038/jid.2012.490

[B36] Korber A Mossner R Renner R Sticht H Wilsmann-Theis D Schulz P Sticherling M Traupe H Huffmeier U Mutations in IL36RN in Patients with Generalized Pustular Psoriasis J Invest Dermatol 2013 doi:10.1038/jid.2013.214. [Epub ahead of print] 10.1038/jid.2013.214 23648549

[B37] Navarini AA Valeyrie-Allanore L Setta-Kaffetzi N Barker JN Capon F Creamer D Roujeau JC Sekula P Simpson MA Trembath RC Rare Variations in IL36RN in Severe Adverse Drug Reactions Manifesting as Acute Generalized Exanthematous Pustulosis J Invest Dermatol 2013 8 1904 1907 23358093 10.1038/jid.2013.44

[B38] Fung MA Barr KL Current knowledge in inflammatory dermatopathology Dermatol Clin 2012 8 667 684 23021053 10.1016/j.det.2012.06.004

[B39] Griffiths WA Pityriasis rubra pilaris: the problem of its classification J Am Acad Dermatol 1992 8 140 142 1732330 10.1016/s0190-9622(08)80543-9

[B40] Tsoi LC Spain SL Knight J Ellinghaus E Stuart PE Capon F Ding J Li Y Tejasvi T Gudjonsson JE Identification of fifteen new psoriasis susceptibility loci highlights the role of innate immunity Nat Genet 2012 8 1341 1348 23143594 10.1038/ng.2467 PMC3510312

[B41] Ammar M Bouchlaka-Souissi C Zaraa I Helms C Doss N Bouazizi F Dhaoui R Ossman AB Ammar-el Gaied AB, Mokni M: **Family-based association study in Tunisian familial psoriasis** Int J Dermatol 2012 8 1329 1334 23067081 10.1111/j.1365-4632.2012.05523.x

[B42] Fuchs-Telem D Sarig O Van Steensel MA Isakov O Israeli S Nousbeck J Richard K Winnepenninckx V Vernooij M Shomron N Familial pityriasis rubra pilaris is caused by mutations in CARD14 Am J Hum Genet 2012 8 163 170 22703878 10.1016/j.ajhg.2012.05.010 PMC3397268

[B43] Jordan CT Cao L Roberson ED Pierson KC Yang CF Joyce CE Ryan C Duan S Helms CA Liu Y PSORS2 is due to mutations in CARD14 Am J Hum Genet 2012 8 784 795 22521418 10.1016/j.ajhg.2012.03.012 PMC3376640

[B44] Chernosky ME Porokeratosis: report of twelve patients with multiple superficial lesions South Med J 1966 8 289 294 5910629 10.1097/00007611-196603000-00011

[B45] Rouhani P Fischer M Meehan S Pomeranz MK Disseminated superficial actinic porokeratosis Dermatol Online J 2012 8 24 23286814

[B46] Skupsky H Skupsky J Goldenberg G Disseminated superficial actinic porokeratosis: a treatment review J Dermatolog Treat 2012 8 52 56 20964569 10.3109/09546634.2010.495381

[B47] Kaur S Thami GP Mohan H Kanwar AJ Co-existence of variants of porokeratosis: a case report and a review of the literature J Dermatol 2002 8 305 309 12081163 10.1111/j.1346-8138.2002.tb00268.x

[B48] Zhou Y Liu J Fu X Yu Y Shi B Yu G Shi Z Wu W Pan F Tian H Identification of three novel frameshift mutations of the MVK gene in four Chinese families with disseminated superficial actinic porokeratosis Br J Dermatol 2013 8 193 195 23834120 10.1111/bjd.12224

[B49] Zhang SQ Jiang T Li M Zhang X Ren YQ Wei SC Sun LD Cheng H Li Y Yin XY Exome sequencing identifies MVK mutations in disseminated superficial actinic porokeratosis Nat Genet 2012 8 1156 1160 22983302 10.1038/ng.2409

[B50] Schafer BL Bishop RW Kratunis VJ Kalinowski SS Mosley ST Gibson KM Tanaka RD Molecular cloning of human mevalonate kinase and identification of a missense mutation in the genetic disease mevalonic aciduria J Biol Chem 1992 8 13229 13238 1377680

[B51] Drenth JP Cuisset L Grateau G Vasseur C van de Velde-Visser SD De Jong JG Beckmann JS van der Meer JW Delpech M Mutations in the gene encoding mevalonate kinase cause hyper-IgD and periodic fever syndrome. International Hyper-IgD Study Group Nat Genet 1999 8 178 181 10369262 10.1038/9696

[B52] Houten SM Kuis W Duran M De Koning TJ Van Royen-Kerkhof A Romeijn GJ Frenkel J Dorland L De Barse MM Huijbers WA Mutations in MVK, encoding mevalonate kinase, cause hyperimmunoglobulinaemia D and periodic fever syndrome Nat Genet 1999 8 175 177 10369261 10.1038/9691

[B53] Arima K Kinoshita A Mishima H Kanazawa N Kaneko T Mizushima T Ichinose K Nakamura H Tsujino A Kawakami A Proteasome assembly defect due to a proteasome subunit beta type 8 (PSMB8) mutation causes the autoinflammatory disorder, Nakajo-Nishimura syndrome Proc Natl Acad Sci U S A 2011 8 14914 14919 21852578 10.1073/pnas.1106015108 PMC3169106

[B54] Liu Y Ramot Y Torrelo A Paller AS Si N Babay S Kim PW Sheikh A Lee CC Chen Y Mutations in proteasome subunit beta type 8 cause chronic atypical neutrophilic dermatosis with lipodystrophy and elevated temperature with evidence of genetic and phenotypic heterogeneity Arthritis Rheum 2012 8 895 907 21953331 10.1002/art.33368 PMC3278554

[B55] Compassionate Use Treatment Protocol I4V-MC-JAGA Treatment of Autoinflammatory Syndromes Expected to Benefit from JAK Inhibition http://clinicalstudies.info.nih.gov/cgi/wais/bold032001.pl?A_12-AR-8001.html@CANDLE@@@@

[B56] Agarwal AK Xing C DeMartino GN Mizrachi D Hernandez MD Sousa AB Martinez DeVillarreal L DosSantos HG Garg A PSMB8 encoding the beta5i proteasome subunit is mutated in joint contractures, muscle atrophy, microcytic anemia, and panniculitis-induced lipodystrophy syndrome Am J Hum Genet 2010 8 866 872 21129723 10.1016/j.ajhg.2010.10.031 PMC2997366

[B57] Kanazawa N Nakajo-Nishimura syndrome: an autoinflammatory disorder showing pernio-like rashes and progressive partial lipodystrophy Allergol Int 2012 8 197 206 22441638 10.2332/allergolint.11-RAI-0416

[B58] McDermott MF Aksentijevich I Galon J McDermott EM Ogunkolade BW Centola M Mansfield E Gadina M Karenko L Pettersson T Germline mutations in the extracellular domains of the 55 kDa TNF receptor, TNFR1, define a family of dominantly inherited autoinflammatory syndromes Cell 1999 8 133 144 10199409 10.1016/s0092-8674(00)80721-7

[B59] Toplak N Frenkel J Ozen S Lachmann HJ Woo P Kone-Paut I De Benedetti F Neven B Hofer M Dolezalova P An international registry on autoinflammatory diseases: the Eurofever experience Ann Rheum Dis 2012 8 1177 1182 22377804 10.1136/annrheumdis-2011-200549

[B60] Ozen S Frenkel J Ruperto N Gattorno M The Eurofever Project: towards better care for autoinflammatory diseases Eur J Pediatr 2011 8 445 452 21360011 10.1007/s00431-011-1411-z

